# Early Confirmation of *Mycoplasma pneumoniae* Infection by Two Short-Term Serologic IgM Examination

**DOI:** 10.3390/diagnostics11020353

**Published:** 2021-02-20

**Authors:** Ha Eun Jeon, Hyun Mi Kang, Eun Ae Yang, Hye Young Han, Seung Beom Han, Jung Woo Rhim, Kyung-Yil Lee

**Affiliations:** 1 Department of Pediatrics, College of Medicine, The Catholic University of Korea, 222, Banpo-daero, Seocho-gu, Seoul 06591, Korea; haeun_bu@naver.com (H.E.J.); pedhmk@gmail.com (H.M.K.); unaver@hanmail.net (H.Y.H.); beomsid@catholic.ac.kr (S.B.H.); benign7@hanmail.net (J.W.R.); leekyungyil@catholic.ac.kr (K.-Y.L.); 2 Department of Pediatrics, The Catholic University of Korea, Daejeon St. Mary’s Hospital, 64 Daeheung-ro, Jung-gu, Daejeon 34943, Korea; 3 Junglock Biomedical Institute, Daejeon, 127, Yuchun-ro, Jung-gu, Deajeon 34886, Korea

**Keywords:** children, diagnosis, ELISA, microparticle agglutination, *Mycoplasma pneumoniae*, polymerase chain reaction

## Abstract

The aim of the present study is to re-evaluate the clinical application of two-times serologic immunoglobulin M (IgM) tests using microparticle agglutination assay (MAA), an enzyme-linked immunosorbent assay (ELISA), and polymerase chain reaction (PCR) assay in diagnosing *Mycoplasma pneumoniae* (MP) infection. A retrospective analysis of 62 children with MP pneumonia during a recent epidemic (2019–2020) was conducted. The MAA and ELISA immunoglobulin M (IgM) and IgG measurements were conducted twice at admission and around discharge, and MP PCR once at presentation. Diagnostic rates in each test were calculated at presentation and at discharge. The seroconverters were 39% (24/62) of patients tested by MAA and 29% (18/62) by ELISA. At presentation, the diagnostic positive rates of MAA, ELISA, and PCR tests were 61%, 71%, and 52%, respectively. After the second examination, the rates were 100% in both serologic tests. There were positive correlations between the titers of MAA and the IgM values of ELISA. The single serologic IgM or PCR tests had limitations to select patients infected with MP in the early stage. The short-term, paired IgM serologic tests during hospitalization can reduce patient-selection bias in MP infection studies.

## 1. Introduction

*Mycoplasma pneumoniae* (MP) is one of the major respiratory pathogens that cause community-acquired pneumonia in children and young adults [1]. MP pneumonia epidemics have occurred with three- to four-year cycles in South Korea during recent decades, and the most recent epidemic occurred in 2019 [2,3]. MP pneumonia is a self-limiting disease, and the majority of the patients recover from the disease without complications. However, some children, especially older children, have intractable or refractory MP pneumonia, in which fever persists and pneumonia progression and higher laboratory values, including C-reactive protein and procalcitonin shown in community-acquired pneumonia, are manifested [4,5,6,7,8].

The rapid diagnosis of MP pneumonia is crucial for determining treatment options for severe cases. Although polymerase chain reaction (PCR) assays are a rapid and effective test for early diagnosis, the sensitivity and specificity of PCR assays are influenced by many confounding factors such as different detection rates regardless of the disease severity and disease stage, age of the patients, sampling sites, causative strain subtypes, and the technical errors [9,10,11]. Furthermore, long- term carriers exist in MP epidemics [12,13,14,15].

Serology tests are commonly used as diagnostic tools for infectious disease, including MP infections, and antibody testing methods include indirect microparticle agglutination assay (MAA) and enzyme-linked immunosorbent assay (ELISA) [14,15]. MAA is used to identify the presence of MP immunoglobulin M (IgM) antibodies and has widely been used in East Asian countries including Japan, China, and Korea [16]. It has been proposed that serologic confirmation of acute infection is to detect a four-fold or greater increase in IgG levels at intervals of two weeks or longer [14]. This approach is difficult to use as a criterion for patient selection in clinical studies. Thus, many researchers have used single IgM levels with or without PCR assay, and a positive IgM titer, especially a high titer, is regarded as an acute infection. Ever since the nationwide MP epidemic in 2003, a two short-term assessment of serologic IgM titers by MAA for the selection of patients with acute infections has been used, and this method has been clinically more reliable in reducing selection-bias in MP studies [17,18,19,20,21].

The purpose of this study was to compare the two serologic testing methods—MAA and ELISA titers—by investigating the correlation between MAA and the ELISA titers and understanding clinical implications of the results of subsequent IgM titers followed up in short interval durations.

## 2. Methods

### 2.1. Study Participants

This was a retrospective cohort study of patients below 15 years old who were admitted at The Catholic University of Korea Daejeon St. Mary’s Hospital for MP pneumonia during the recent 2019 nationwide epidemic. Patients with pneumonia admitted from May 2019 to February 2020 were included in this study. The inclusion criteria of this study were as follows: (1) underwent at least two MP serology tests including IgM and IgG using the MAA (Serodia-Myco II, Fujirebio, Tokyo, Japan) and ELISA (Chorus MP IgM and IgG ELISA, Diesse Diagnostica, Senese, Siena, Italy) methods in intervals of at least 48 h between the two tests, (2) additionally received bacterial PCR assay at admission, and (3) cases were satisfied with criteria in MAA method above fit the definition for MP pneumonia. Patients that fit all of the criteria above were included in this study. The exclusion criteria were as follows: (1) patients that only had initial serology test results, (2) patients that only had one type of MP serology testing methods (MAA or ELISA), (3) patients that did not undergo bacterial PCR assay upon admission, and (4) patients that had chronic underlying diseases that predisposed them to have recurrent pulmonary infections.

### 2.2. Study Definitions

The diagnostic criteria for acute MP pneumonia differed for each of the testing methods. In serology testing using the MAA method, MP pneumonia was defined using the following criteria: (1) negative to positive IgM seroconversion (≥1:40), (2) two-fold or greater increase in IgM titers tested in intervals of at least 48 h or more, or (3) initial and continuous high titers ≥1:640 at follow up. Patients whose serology did not show increasing IgM titers or those that showed decreased titers at follow-up were regarded as false positives, following previous studies at our center [18,19,20,21]. In this study, IgM titer was calculated to 1:1280, and high titers of >1:1280 were reported as ≥1280. For IgM serology testing using the ELISA method, the cut-off values for interpreting MP infection status were used according to the instructions of the manufacturer—positive (IgM > 1.1), negative (IgM < 0.9), and equivocal (IgM 0.9–1.1). For IgG, the cut-off values were as follows: positive (IgG > 18 AU/mL), negative (IgG < 12 AU/mL), and equivocal (12–18 AU/mL).

### 2.3. Study Design

After applying the inclusion and exclusion criteria, the final study participants were determined, and their electronic medical records were reviewed for the following data: demographics, clinical and image findings, and laboratory results. Upon admission, all patients received bacterial PCR assay detecting MP, *Streptococcus pneumoniae*, *Haemophilus influenzae*, *Legionella pneumoniae*, *Chlamydia pneumoniae*, *Bordetella pertussis* via CFX96 Touch TM Real-Time PCR detection system (Bio-Rad, Laboratories, Inc., Hercules, CA, USA). The respiratory specimen used for bacterial PCR assay were obtained via nasopharyngeal swab.

The positive rates of the two serology testing methods and PCR assay at presentation and at discharge were investigated. Using the paired serology results (initial and follow-up) from each of the patients, IgM and IgG titers of MAA and ELISA were compared.

### 2.4. Statistical Analysis

All calculations were performed using SPSS ver. 14.0 (SPSS Inc., Chicago, IL, USA). The data are expressed as mean ± standard deviation (SD) for continuous variables or as the number of cases (percentage) of a specific group for categorical variables. Comparisons of titers between MAA and ELISA were performed by Pearson’s correlation. All *p*-values were two-tailed, and *p* values of <0.05 were considered statistically significant.

## 3. Results

### 3.1. Demographic, Clinical, and Laboratory Findings

A total of 62 patients who had two paired serology results (initial and follow-up) for both the MAA and ELISA methods and results for bacterial PCR assay were included in the study (Table 1). The mean age of the study participants was 6.4 ± 2.9 years old (range 1–14 years old). The male-to-female ratio was 1:1 (31:31). The preadmission and total fever durations were 5.0 ± 2.6 days and 5.6 ± 3.0 days, respectively. The duration of admission was 6.1 ± 1.7 days, and the mean interval of the two MP serologic tests obtained from each of the patients was 3.2 ± 0.9 days. All patients were treated with macrolides and corticosteroids within 24–36 h, as reported in a previous study [18].

The results of the IgM serology using the MAA method (*n* = 62) were as follows: negative to positive seroconversion was observed in 24 (38.7%) patients, increased ≥two-fold titers were observed in 27 (43.5%) patients, and high titers ≥1:640 in both initial and follow-up serology testing were observed in 11 (17.7%) patients. The results of their IgM serology using the ELISA method were as follows: A total of 44 (71.0%) patients were seropositive at the initial examination, 12 (19.3%) were seronegative, and 6 (9.7%) were equivocal. At follow-up, all 62 patients became seropositive for IgM; increased IgM values were observed in 54 (87.1%) of the patients, 5 (8.1%) showed decreased IgM values, and 3 (4.8%) showed no change in IgM values (Table 2). The IgG serology test results using the ELISA method for these patients were as follows: A total of 44 (71.0%) patients were initially IgG seronegative and 18 (29.0%) were initially seropositive. At follow-up, among the 44 initially IgG seronegative patients, 45.5% (20/44) showed positive seroconversion, 20.5% (9/44) showed negative to equivocal values, and 34.1% (15/44) remained IgG seronegative.

A total of 52% (32/62) of the patients were positive for MP in the bacterial PCR assay. Among them, 40.6% (13/32) of the cases showed co-detection with other bacterial species such as *H. influenzae* and *S. pneumoniae*. Overall, the positive rates of the different types of testing methods at the initial examination were 61% (38/62) for MAA, 71% (44/62) for ELISA, and 52% (32/62) for PCR assay. At short-term follow-up serology testing, both MAA and ELISA methods showed 100% seropositive and met the diagnostic criteria for MP pneumonia (Table 1).

### 3.2. Comparison between MAA and ELISA

The patients were divided into three groups according to the MAA criteria for the diagnosis of acute MP pneumonia, and in each of the groups, the serology results of the ELISA tests were compared. Group 1 included 24 patients that showed negative to positive seroconversion (from negative to ≥1:40~ ≥1:1280). Group 2 included 27 patients that showed increased IgM titers (19 patients increased ≥four-fold), and group 3 included 11 patients that had high IgM titers (≥1:640) at initial and follow-up tests. In group 1, 50% (12/24) were initially IgM seronegative, 16.7% (4/24) initially equivocal, and 33.3% (8/24) initially seropositive according to the ELISA testing method. At follow-up, all patients became IgM seropositive, however, one case showed slightly decreasing positive titers. In group 2, 92.6% (25/27) and 7.4% (2/25) were initially IgM seropositive and equivocal by the ELISA, and at follow-up, all patients showed IgM seropositive, but one patient’s IgM value did not increase. In group 3, all were initially seropositive; 45.5% (5/11) patients showed increasing IgM values, 36.4% (4/11) showed positive but decreasing values, and 18.2% (2/11) showed the same positive IgM values (Table 2).

**Figure 1:**
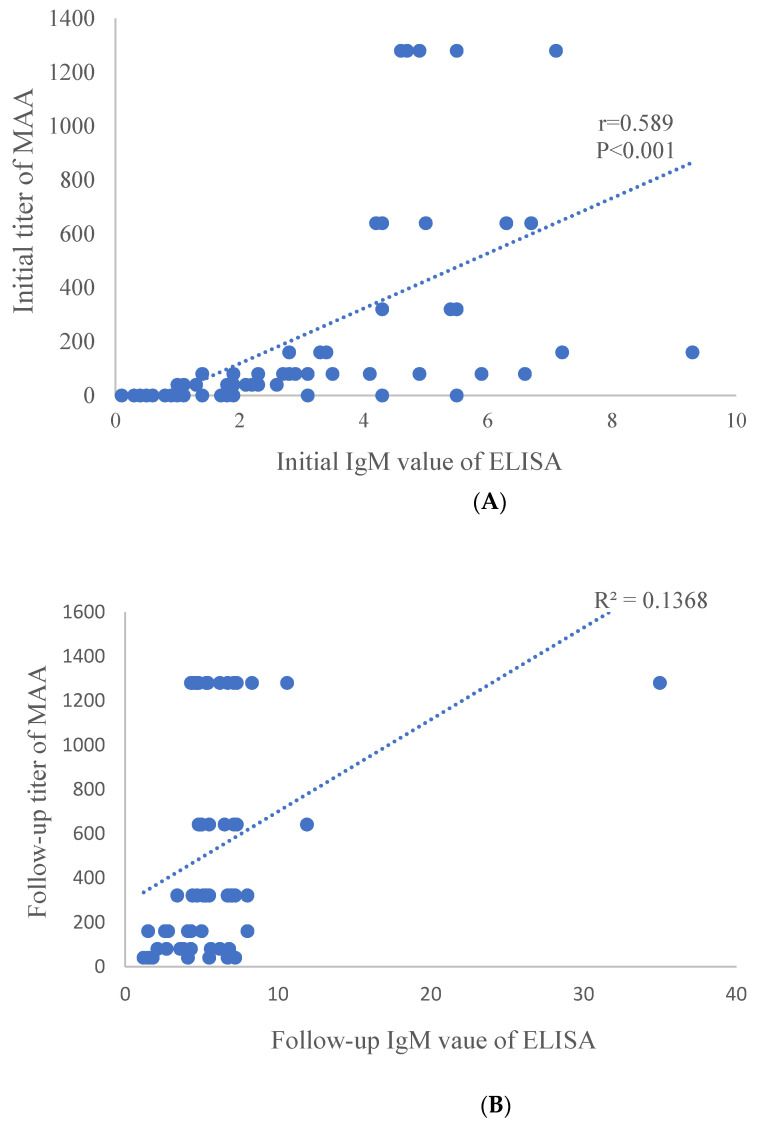
Correlation between IgM of MAA and ELISA. Initial IgM (**A**) and later IgM (**B**) showed significant correlation between MAA and ELISA (*p* < 0.001 and *p* = 0.003). IgM titer was calculated to 1:1280, and high titers of ≥1:1280 were expressed as 1280 in MAA. r is Pearson’s correlation. Abbreviations: MAA, microparticle hemagglutinin assay; ELISA, enzyme-linked immunosorbent assay.

The relationship between IgM titers by the MAA method and IgM values by the ELISA method was investigated. A linear regression model showed a significant positive correlation between the initial IgM value by the ELISA method and the initial IgM titer by the MAA method (*p* < 0.001, R^2^ = 0.347) (Figure 1A). Moreover, a significant positive correlation was observed between the follow-up IgM values by the ELISA method and IgM titers by the MAA method (*p* = 0.003, R^2^ = 0.138) (Figure 1B). However, a stronger correlation was observed in the initial test compare to the follow-up test.

## 4. Discussion

MP is one of the major pathogens causing respiratory tract infections in childhood, with other pathogens including influenza virus and coronavirus. In these infections, pathogen-specific IgM antibodies are produced first, and then specific IgG and IgA antibodies follow during systemic immune responses to the insults of the pathogen [22,23]. Accordingly, there is a time-gap of several days or longer between the onset of symptoms such as fever, sore throat, myalgia, and pneumonia and the appearance of pathogen-specific IgM antibodies. In this study, we found that approximately a third of the patients were seronegative for MP-specific IgM at presentation, reported in our previous studies [18,19,20,21]. In addition, higher rates of IgG seronegativity were observed compared to IgM, and the seroconversion of IgG occurred later than that the seroconversion of IgM during the early stage of MP infection. It is natural that patients that visit the hospital during the early stage of disease presentation tend to show a higher rate of negative or lower titers of IgM and IgG [20]. However, some patients can show a longer time to seroconversion in MP infection as seen in SARS and COVID-19 [24,25]. Previously, we found that late-seroconverters may have a tendency toward a more severe form of pneumonia [18]. Therefore, a single IgM serologic test has limitations in selecting patients that visit the hospital during the early stage of disease presentation and/or those that have a more severe form of pneumonia in the early stage. Furthermore, some patients can show long-term IgM positivity after MP infection [26]. Therefore, patients with pneumonia caused by other pathogens may be misdiagnosed as MP pneumonia due to seropositive MP IgM levels, especially during MP epidemics and post-epidemic periods.

The positive rates in detecting IgM can vary across serologic methods because the antigens used in serologic kits and assays differ. Thus, the sensitivity and specificity of IgM/IgG are somewhat different according to the used kits. Although we used MAA as the “gold standard” in this study, we found that ELISA IgM/IgG kit used in this study may have a higher sensitivity than MAA because of lower negativity in the early stage of disease presentation. In the present study, at admission, the positive rates of MAA, ELISA, and PCR assay were 61%, 71%, and 52%, respectively. In contrast, the positive rates of paired IgM tests were 100% in both serologic methods. However, little change or decreased IgM values obtained from a short-term follow-up of ELISA IgM, especially lower values, may depict recent past infection. However, paired IgG values could in part help differentiating acute infection from the recent past infection because some patients reach the peak levels of IgM at presentation, and IgG seroconversion began to occur in the early stages and IgG values continuously elevated during the early convalescent stage of MP infection.

The IgM titers in MAA were positively correlated with the numerical values in ELISA. We previously reported that there was a positive correlation between titers in MAA and titers of cold agglutinins, which are nonspecific IgM and are used for the diagnosis of MP infection in the era of the absence of MP-specific serologic assays [20]. We have learned that the serologic tests reflect a systemic immune reaction to MP infection correctly, but there are somewhat differences in titer patterns in individuals between the serologic kits as shown in this study. It is possible that during MP infection, lots of antigens are produced, and the kinds and amounts of antigens, in addition to the immune function may vary on an individual basis. It is recommended that paired specific IgG serologic tests two to four weeks apart are needed to confirm MP infection [14]. However, this policy has difficulties in selecting patients and is not helpful in clinical practice.

Many study groups have reported the sensitivity and specificity of serologic methods through comparison with PCR assay as the “gold standard” [27,28]. Indeed, it has been observed that a part of patients with MP pneumonia show PCR negative at presentation, even though new PCR assays have been introduced with high PCR sensitivity and specificity [29,30]. We have experienced that PCR positive rates were far lower than single initial IgM tests, especially in children [18,19,31]. In addition, in this study, we found that PCR positivity was not associated with preadmission fever duration, although our sample size was small. These negative findings may be in part due to specimen quality and collection method, however, may be explained that MP itself is not a direct cause of lung injury, but other smaller substances and corresponding immune reactions may be responsible for pneumonia and extrapulmonary manifestations in MP infection.

The immunopathogenesis of MP infection and other pathogen infections, including COVID-19, remains unknown. It is proposed that the etiological substances in infectious diseases may be produced from certain pathogen-infected cells, including MP infection [21,32,33]. If MP or acute respiratory distress syndrome coronavirus-2 (SARS-COV-2) itself is responsible for lung injury, researchers should prove the pathogen using the materials from lung tap and/or pleural effusion not using the materials from upper or lower respiratory tracts such as throat swab or bronchial lavage fluid, through culture method, not PCR. PCR assays cannot differentiate infection and colonization [34] and cannot provide definitive evidence of the host’s systemic immune reaction to MP.

The limitations of this study include the retrospective design and small sample size.

Because there is no standardized serologic test available, we used MAA as the “gold standard”. However, this study provides novel insight on serological diagnostic methods through a comparison of two serologic tests.

## 5. Conclusions

In conclusion, a PCR test and/or single IgM test have limitations for confirmative diagnosis and patient selection on MP infection; there are PCR negative patients, especially those that were examined at late disease course (false negative) and MP colonized patients (carriers) with pneumonia caused by other pathogens during MP epidemics (false positive). In addition, early presented patients and/or severe MP pneumonia show IgM seronegative during the early stages of infection (false negative), and other pneumonia patients with prolonged seropositivity caused by prior MP infection show IgM seropositive (false positive). Thus, short-term, follow-up, or repeated IgM (and IgG) serologic tests during hospitalization are helpful for reducing patient-selection bias in MP infection studies.

## Figures and Tables

**Table 1:** Positive rates of *Mycoplasma* diagnostic tests during hospitalization (*n* = 62).

	**Positive Rates, *n* (%)**	
**Testing Method**	**At Presentation**	**At Discharge**
MAA *	38 (61)	62 (100)
ELISA	44 (71)	62 (100)
PCR	32 (52)	ND

* Patients with (1) negative to positive immunoglobulin M (IgM) seroconversion (≥1:40), (2) two-fold or greater increase in IgM titers tested in intervals of at least 48 h or more, or (3) initial and continuous high titers >1:640 at follow-up were considered positive. Abbreviations: MAA, microparticle hemagglutinin assay (Serodia Myco II); ELISA, enzyme-linked immunosorbent assay (Chorus MP IgM and IgG ELISA); PCR, polymerase chain reaction; ND, not determined.

**Table 2:** Changes of antibody status during the short-term period.

Positive Results, n (%)				
MAA (*n* = 62)		ELISA (*n* = 62)		
		1st Examination		2nd Examination
Seroconversion	24 (39)	IgM negative	12	All IgM positive, but 1 case decreased value
(negative→ ≥1:40)		IgM equivocal	4	
		IgM positive	8	
Increased titer	27 (44)	IgM equivocal	2	All IgM positive (26 cases increases and 1 case showed same value)
		IgM positive	25	
Both high titer of ≥1:640	11 (18)	IgM positive	11	5 cases increase, 4 cases decrease, 2 cases same value
			

The mean duration from the first examination to the second examination was 3.2 ± 0.9 days. Abbreviations: MAA, microparticle hemagglutinin assay; ELISA, enzyme-linked immunosorbent assay.

## Abbreviations

MP	Mycoplasma pneumoniae
MAA	microparticle agglutination assay
ELISA	enzyme-linked immunosorbent assays
PCR	polymerase chain reaction
IgM	immunoglobulin M
